# The genus *Meiothrips* Priesner (Thysanoptera, Phlaeothripidae, Idolothripinae) with a key and a new species from China

**DOI:** 10.3897/zookeys.177.2695

**Published:** 2012-03-23

**Authors:** Li-Hong Dang, Ge-Xia Qiao

**Affiliations:** 1 Key Laboratory of Zoological Systematics and Evolution, Institute of Zoology, Chinese Academy of Sciences, No. 1 Beichen West Road, Chaoyang District, Beijing 100101, P.R.China; 2 Graduate University of Chinese Academy of Sciences, No. 19, Yuquan Road, Shijingshan District, Beijing 100049, P.R.China

**Keywords:** *Meiothrips*, Thysanoptera, Phlaeothripidae, Idolothripinae, new species, key, China

## Abstract

The genus *Meiothrips* Priesneris reviewed, with *Meiothrips fuscicrus***sp. n.**, and *Meiothrips nepalensis* Kudo & Ananthakrishnan recorded and described from China, and a key provided to the five known species. COI sequences of the new species and *Meiothrips nepalensis* are also provided.

## Introduction

*Meiothrips* was erected as a subgenus of *Idolothrips* with the type-species *Meiothrips annulipes* Priesner (1929). This was recognized as a full genus by Bagnall (1934) as a member of the *Idolothrips* – *Actinothrips* group. Subsequently four species have been added to the genus: *Meiothrips menoni* Ananthakrishnan, *Meiothrips nepalensis* Kudo & Ananthakrishnan, *Meiothrips kurosawai* Okajima, and *Meiothrips baishanzuensis* Duan & Li. However, the last of these has recently been recognized as belonging to a different genus, and this information will be published separately. As a result of the new species described below, there are still five species recognized in the genus, of which three are recorded from China.

DNA barcoding sequences (COI) are provided here as identifying characters for *Meiothrips fuscicrus* sp. n., also the newly recorded species *Meiothrips nepalensis*. We suggest that, if possible, DNA barcoding sequences (COI or other molecular marker) should be provided when a new taxon is described in order to provide an additional method of identification.

## Materials and methods

The samples of thrips were collected into 95% ethanol and stored at -20°C. Total genomic DNA was extracted from single thrips using the method of Rugman-Jones et al. (2006), Mound et al. (2010) and Chen et al. (2011). The COI was amplified using primers LCO1490 and HCO2198 (Mound et al. 2010). Sequences were assembled by Seqman II (DNAstar, Inc., Madison, WI, USA) and then aligned using Clustal W.

The remaining carcass was removed and stored in 10% KOH for several days (usually 4–6 days for the large thrips, about 8–10 mm), then mounted in Canada balsam. Descriptions and pictures are based on permanent slides of specimens using a Leica DM4000B microscope. Measurements and pictures were processed using the Leica Microsystems with Microsoft QWIN (Leica QWin plus). Thrips terminology in this paper generally follows Mound (2011) and Okajima (2006). The unit of measurements is micrometre.

### Depositories and abbreviations

All specimens including types and vouchers are deposited in the National Zoological Museum of China, Institute of Zoology, Chinese Academy of Sciences, Beijing, China. The following abbreviations are used for pronotal setae: *am*-anteromarginals, *aa*-anteroangulars, *ml*-midlaterals, *epim*-epimerals, *epima*-epimeral accessory, *pa-*posteroangulars, *pm*-posteromarginals.

### Meiothrips

Priesner

http://species-id.net/wiki/Meiothrips

Idolothrips (Meiothrips) Priesner, 1929: 197. Type-species: *Idolothrips (Meiothrips) annulatus* Priesner, now considered a synonym of *Acanthinothrips annulipes* Bagnall (Palmer and Mound 1978).
Meiothrips Priesner: Bagnall 1934: 494;  1964: 98; Kudo and Ananthakrishnan 1974: 385; Palmer and Mound 1978: 209.

#### Generic diagnosis.

 Body large. Head much longer than width across eyes, prolonged in front of eyes, usually shorter than broad except in one species about twice as long as broad; eyes normal or obviously prolonged on ventral surface; interocellar, postocellar, postocular, mid-dorsal and posterior-dorsal setae usually well developed, sometimes small. Maxillary stylets short and far apart. Antennae 8-segmented, very slender; segment III longest, usually more than twice width across eyes; segments III and IV with 2 and 4 sense-cones. Pronotum major setae usually well developed setae, sometimes *aa* small and epimeral accessory always minute; notopleural sutures incomplete; basantra and ferna present. Mesopraesternum boat-shaped. Metathoracic sternopleural sutures absent. Wings usually fully developed with or without numerous duplicated cilia. All legs normal, femora with several spine-setae. Pelta always broad, lateral lobes broadly joined to median major lobe; abdominal tergites II–VII each with two pairs of sigmoid wing-retaining setae; tergites V–VIII never with lateral tubercles; tube much longer than head, surface with numerous fine setae, sometimes with 2 rows of stout tubercles and many large and small tubercles or denticles on dorsal surface; anal setae much shorter than tube.

#### Distribution.

 China (Zhejiang, Yunnan, Hainan); India, Nepal, Malaysia, Thailand.

#### Biology.

 The species of *Meiothrips* are presumed to all feed only on fungal-spores. In the field, *Meiothrips* natural populations with deposited egg masses have been observed only on newly-dead dry or withered leaves hanging on branches.

#### Comments.

 This genus is close to *Idolothrips* and *Bactrothrips*. The morphological characters of the females, and the head and thorax of males, are similar in the three genera. Mound and Palmer (1983) pointed out that the species are intermediate in structure between *Idolothrips* and *Bactrothrips*, such that each could be placed in a separate genus if the traditional concepts employed in the *Bactrothrips* complex were accepted. *Meiothrips kurosawai* is particularly unusual with the eye prolonged posteriorly on the ventral surface of the head, and a long preocular projection. The systematic position and relationships of these genera require further study.

#### Key to Meiothrips species

**Table:** 

1	Eyes posteriorly prolonged on ventral surface; head with a long preocular projection, about 2 times as long as width	*Meiothrips kurosawai*
–	Eyes posteriorly not prolonged on ventral surface (Figs 2, 13, 20, 23); head with shorter preocular projection, shorter than broad	2
2	Forewing without duplicated cilia	*Meiothrips annulipes*
–	Forewing with duplicated cilia	3
3	Antennal segments VI–VII normal, without a short apical, ventral prolongation; postocular setae small, much shorter than postocular cheek setae; tube of male without dorsal tubercles or denticles	*Meiothrips menoni*
–	Antennal segments VI–VII with a short apical, ventral prolongation; postocular setae similar or longer than postocular cheek setae; tube of male with dorsal tubercles or denticles	3
4	Middle and hind femora bicolored, with about basal half yellow and apical half dark brown	*Meiothrips nepalensis*
–	Middle femora uniformly dark brown, hind femora bicolored, with basal 2/3 and extreme distal parts yellow, the rest dark brown	*Meiothrips fuscicrus* sp. n.

### Meiothrips fuscicrus sp. n.

urn:lsid:zoobank.org:act:06A55A81-94F2-4BD5-A268-FA0753E9709B

http://species-id.net/wiki/Meiothrips_fuscicrus

Figs 1 –19

#### Male macroptera.

 Body uniformly dark brown; antennal segments I–II brown, III yellow but shaded in distal and near basal part, IV–V shaded but yellow in distal pedicels 0.3–0.4, VI–VIII brown; fore wings shaded with brown longitudinal band medially in basal half; femora dark brown but about basal 2/3 and extreme distal of hind femora yellow (Figs 7, 16), tarsi, distal half and extreme bases of tibiae yellow; tube dark brown; major setae yellowish.

**Figures 1–12.**
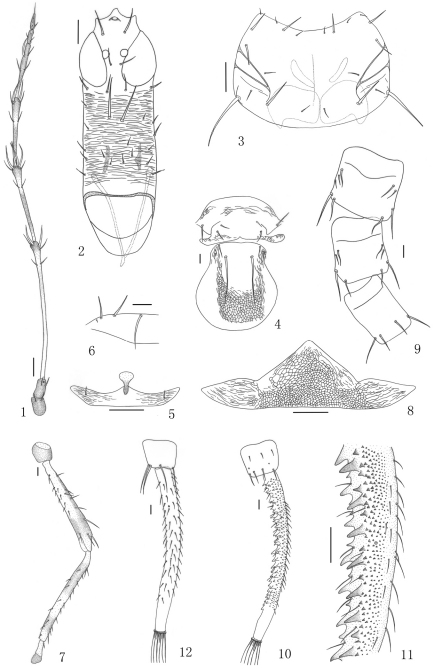
*Meiothrips fuscicrus*sp. n. Male. **1** antenna **2** dorsal view of head **3** dorsal view of pronotum **4** dorsal view of mesonotum and metanotum **5** mesopraesterum **6** base of forewing **7** hind leg **8** pelta **9** dorsal view of abdominal tergites VI–VIII **10** abdominal tergite IX and tube **11** lateral view of tube. Female **12** abdominal tergite IX and tube. Scale bars=100 microns.

**Figures 13–19.**
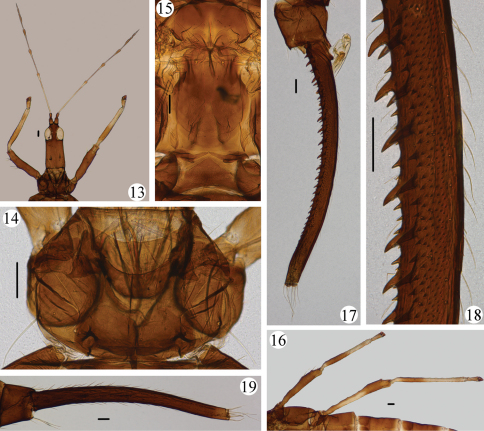
*Meiothrips fuscicrus*sp. n. Male. **13** antenna, head, pronotum and fore legs **14** dorsal view of pronotum **15** dorsal view of mesonotum, metanotum and pelta **16** mid and hind legs **17** abdominal tergite IX and tube **18** lateral view of tube. Female **19** abdominal tergite IX and tube. Scale bars=100 microns.

Head 2.3 times as long as width across eyes, projecting in front of eyes, transversely striate; interocellar setae long, longer than width of one eye, one pair of postocellar setae about 1.7 times as long as diameter of posterior ocellus; eyes developed, about 0.4 of head length, postocular setae and one pair of postocular cheek setae similar with postocellar setae; mid-dorsal setae longest; cheeks with several pairs of minor setae. Maxillary stylets wide apart, retracted into head one fourth way to posterior margin of eyes (Figs 2, 13). Antennal segment III 2.2 times as long as head width across eyes (Figs 1, 13), III with 2 sensoria, IV with 4, V with 2, VI and VII each with one, these sensoria on III and IV about 2.0 times as long as apical width of segment, segments VI–VII with a short apical, ventral prolongation.

Pronotum smooth, anterior margin concave; three pairs of *am*, one pair almost as long as *aa epima* much shorter than one third of longest *epim* (Figs 3, 14); prosternal basantra around the tip of mouth-cone, ferna triangular. Mesopraesternum boat-shaped (Fig. 5). Metanotal median setae well developed, metanotum smooth on anterior third, with weak reticulate sculpture on posterior half (Figs 4, 15); metathoracic sternopleural sutures absent. Fore wings broad, with 42 duplicated cilia.

Pelta with reticulate sculpture slightly longitudinal (Figs 8, 15); abdominal segments VI–VIII without lateral tubercles (Fig. 9); tergite IX setae much shorter than tube; tube about 2.3 times as long as head, weakly constricted near apex, with about 20 pairs of stout tubercles and many small tubercles (Figs 10, 11, 17, 18), laterally with few weak setae. Sternites with irregular transverse row of discal setae, no pore plates.

*Measurements* (male in microns). Body length 8171. Head, length 787; width across eyes 342; interocellar setae 105, postocellar setae 62, diameter of posterior ocellus 36; postocular setae 63, postocular cheek setae 59; mid-dorsal setae 162. Antennal segments III–VIII length (maximum width), 758 (51), 437 (49), 379 (48), 306 (35), 83 (29), 89 (18), sensoria of segment III length 101. Pronotum length (maximum width) 336 (553); setae length, *am* 68, *aa* 67, *ml* 75, *epim* 190, *epima* 26, *pa* 109, *pm* 24. Metanotal median setae length 288. Pelta length 191, width 568. Tergite IX setae S1 141, S2 130. Tube length 1876, stout tubercles length 50, anal setae length 337.

#### Female macroptera.

 Similar to male but larger. Tube smooth with many prominent lateral setae (Figs 12, 19).

*Measurements* (female in microns). Body length 8864. Head, length 779; width across eyes 344; interocellar setae 102, postocellar setae 64, diameter of posterior ocellus 35; postocular setae 88, postocular cheek setae 77; mid-dorsal setae 134. Antennal segments III–VIII length (maximum width), 748 (47), 444 (52), 367 (48), 292 (41), 94 (31), 98 (27), sensoria of segment III length 90. Pronotum length (maximum width) 304 (546); setae length, am 51, *aa* 63, *ml* ?, *epim* 185, *epima* 50, *pa* 142. Metanotum median setae length 304. Pelta length 189, width 667. Tergite IX setae S1 255, S2 320. Tube length 1913, anal setae length 367.

#### Specimens examined

. Holotype male: China, Yunnan province, coll. M.Y. Lin by shaking withered tree leaves, 7.x.2010 (slides No.JM10058-9). *Paratypes*: 6 females and 1 male, data as for holotype (slides Nos. JM10058-1, JM10058-5, JM10058-7, JM10058-13, JM10058-15, JM10058-16, JM10058-17).

#### Remarks.

 This species is similar to *Meiothrips nepalensis* in that the tube of males bears about two rows of stout tubercles also many small tubercles. But the new species can be distinguished by the following characters: fore and mid-femora uniformly dark brown, only hind femora bicolored with about basal 2/3 and extreme distal parts yellow (*nepalensis*: fore femora uniformly dark brown, mid- and hind femora bicolored with about basal half yellow and apical half dark brown), pronotal *am* and *aa* setae about 0.3 times as long as *epim* setae (*nepalensis*: *am* and *aa* minute, much less than 0.3 times as long as *epim*).

#### Etymology.

 This species name is composed of two Latin words, “*fuscus* (= brown)” and “*crus* (= leg)”, based on mid-femora uniformly dark brown.

#### COI sequences.

 We received two sequences of the new species, which include 674bp and 647bp with the GenBank numbers JQ411299 and JM411300, respectively.

### Meiothrips menoni

Ananthakrishnan, 1964

http://species-id.net/wiki/Meiothrips_menoni

Figs 20–22

Meiothrips menoni Ananthakrishnan 1964: 99; 1971: 203; 1973: 111; Palmer and Mound 1978: 210.

#### Material examined.

 8 females and 15 males: China, Yunnan Province, coll. Y.F. Han from withered tree leaves, 17–20.xi.1988 (slides Nos. 14214–14222, 14399), and 27.iii–22.iv.1997 (slides Nos. 21026–21033, 22279, 22795–22796, 22443); 1 male: China, Hainan Province, coll. W.Q. Zhang from withered tree leaves, 5.iv.1984.

#### Distribution.

 China (Yunnan, Hainan); India, Thailand, Malaysia.

#### Comments.

 This species is mainly distributed in South Asia. It was described originally from India by Ananthakrishnan (1964) from only one female. The specimens in this study are very similar to the original description, but the mid- and hind tibiae are similar to specimens from Malaysia and Thailand with a dark brown sub-basal mark and a narrow paler brown subapically. Palmer and Mound (1978) discussed the differences of the specimens from India, Malaysia and Thailand. The specimens in this study with long and pale pronotal major setae and head setae are more similar to the Indian specimens than the specimens from Malaysia and Thailand with short and dark setae, although specimens from Thailand have long pronotal *pa* and *am*. Further studies are needed combining the morphological and molecular evidence.

**Figures 20–25.**
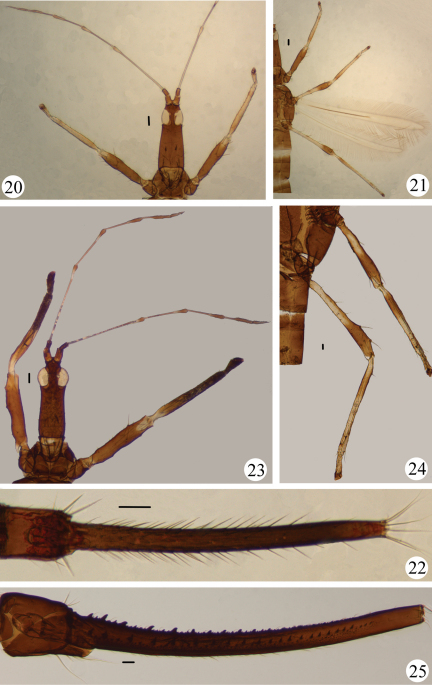
*Meiothrips* spp. **20–22** *Meiothrips menoni*. Male **20** antenna, head, pronotum and fore legs **21** legs **22** abdominal tergite IX and tube **23–25** *Meiothrips nepalensis*. Male **23** antenna, head, pronotum and fore legs **24** mid- and hind legs **25** abdominal tergite IX and tube. Scale bars=100 microns.

### Meiothrips nepalensis

Kudo & Ananthakrishnan, 1974

http://species-id.net/wiki/Meiothrips_nepalensis

Figs 23–25

Meiothrips nepalensis Kudo and Ananthakrishnan 1974: 385; Kudo 1975: 421; Palmer and Mound 1978: 212.

#### Material examined.

 1 female and 2 males: China, Yunnan province, coll. M.Y. Lin by shaking withered tree leaves, 7.x.2010 (slides numbers, JM10058-4, JM10058-8, JM10058-12).

#### Distribution.

 China (Yunnan); Nepal, Thailand.

#### COI sequences. 

It includes 659bp with the GenBank number JQ411298.

#### Comments.

 This species is known from Nepal (Kudo and Ananthakrishnan 1974) and Thailand (Palmer and Mound 1978), and is here recorded from China for the first time. The remarkable structure of this species is that the tube of the male bears two rows of stout tubercles and numerous small and large tubercles. *Idolothrips dissimilis* from Australia also shows similar structures.

## Supplementary Material

XML Treatment for Meiothrips

XML Treatment for Meiothrips fuscicrus

XML Treatment for Meiothrips menoni

XML Treatment for Meiothrips nepalensis
